# Efficacy of a modified remotivational program for persons with Schizophrenia in community mental health center

**DOI:** 10.1192/j.eurpsy.2025.1592

**Published:** 2025-08-26

**Authors:** J. Hsiao, A.-W. Pan

**Affiliations:** 1 Taipei city hospital; 2 National Taiwan University, Taipei, Taiwan, Province of China

## Abstract

**Introduction:**

Patients with schizophrenia often experience deficits in motivation, which can significantly impact their functional abilities and participation in daily life activities as the illness progresses. Motivational deficit is a key concern in the treatment and rehabilitation of schizophrenia. The remotivational program, based on the Model Of Human Occupation, is a therapeutic intervention aimed at improving the volition subsystem and addressing motivational deficit in patients.

**Objectives:**

(1) The modified remotivational program can improve the motivation deficits, social functioning, social skills, and alleviate the psychiatric symptoms of individuals with schizophrenia in the community. (2)The experimental group, compared to the control group, can improve the motivation deficits, social functioning, social skills, and symptoms of patients.

**Methods:**

This study is conducted at a psychiatric specialty hospital in northern Taiwan and recruits participants from a community rehabilitation center. This study included a total of 38 participants, diagnosed with schizophrenia or schizoaffective disorder. The experimental group receives a modified remotivational program twice a week for 50 minutes per session over eight sessions. This experiment employs a randomized assignment and single-blind design, with assessors unaware of group assignments. Statistical analyses involve paired sample t-tests to compare within-group differences pre- and post-intervention, and independent sample t-tests to compare between-group differences post-intervention.

**Results:**

The experimental group underwent a four-week intervention consisting of eight sessions of themodified remotivational program. Results indicated significant improvement in addressing motivational deficits in patients (t=2.61, p=0.01). However, other outcome measures showed no significant differences between the experimental and control groups.The experimental group with average satisfaction ratings ranging from 4.12 to 4.43, and average self-assessment scores ranging from 4.12 to 4.25. Clinical feedback and observations revealed that participants in the experimental group expresses higher interest and engagement in the modified remotivational program.

**Conclusions:**

While individuals with schizophrenia undergoing community-based mental rehabilitation generally exhibit stable psychiatric symptoms, they often overlook the pervasive impact of negative symptoms, particularly the widespread influence of motivational deficits. This study’s findings demonstrate that the intervention program effectively addresses motivational issues. Therefore, it is recommended that future clinical practices integrate and utilize such interventions to provide community-based psychiatric care with individualized and diverse intervention strategies.

**Disclosure of Interest:**

None Declared

